# Pattern of lymph node spread in gastric cancer: Western multicenter retrospective study

**DOI:** 10.1093/bjsopen/zrag076

**Published:** 2026-07-03

**Authors:** Francesca Blasa, Giuseppe Verlato, Hidde Overtoom, Martina Hermez Chole, Fabrizio Tedone, Federica Filippini, Markos Despotidis, Evgenia Mela, Tania Triantafyllou, Dimitrios Schizas, Dimitrios Theodorou, Magnus Nilsson, Suzanne S Gisbertz, Maria Bencivenga, Ioannis Rouvelas

**Affiliations:** General and Upper GI Surgery Division, Department of Surgery, University of Verona, Verona, Italy; Department of Diagnostics and Public Health, University of Verona, Verona, Italy; Department of Surgery, Amsterdam UMC Location University of Amsterdam, Amsterdam, the Netherlands; Cancer Treatment and Quality of Life, Cancer Center Amsterdam, Amsterdam, the Netherlands; Department of Upper Abdominal Surgery, Center for Digestive Diseases, Karolinska University Hospital, Stockholm, Sweden; Division of Surgery and Oncology, Department of Clinical Science, Intervention and Technology (CLINTEC), Karolinska Institutet, Stockholm, Sweden; General and Upper GI Surgery Division, Department of Surgery, University of Verona, Verona, Italy; General and Upper GI Surgery Division, Department of Surgery, University of Verona, Verona, Italy; First Department of Surgery, National and Kapodistrian University of Athens, Laikon General Hospital, Athens, Greece; First Propaedeutic Department of Surgery, National and Kapodistrian University of Athens, Hippokration General Hospital, Athens, Greece; First Propaedeutic Department of Surgery, National and Kapodistrian University of Athens, Hippokration General Hospital, Athens, Greece; First Department of Surgery, National and Kapodistrian University of Athens, Laikon General Hospital, Athens, Greece; First Propaedeutic Department of Surgery, National and Kapodistrian University of Athens, Hippokration General Hospital, Athens, Greece; Department of Upper Abdominal Surgery, Center for Digestive Diseases, Karolinska University Hospital, Stockholm, Sweden; Division of Surgery and Oncology, Department of Clinical Science, Intervention and Technology (CLINTEC), Karolinska Institutet, Stockholm, Sweden; Department of Surgery, Amsterdam UMC Location University of Amsterdam, Amsterdam, the Netherlands; Cancer Treatment and Quality of Life, Cancer Center Amsterdam, Amsterdam, the Netherlands; General and Upper GI Surgery Division, Department of Surgery, University of Verona, Verona, Italy; Department of Upper Abdominal Surgery, Center for Digestive Diseases, Karolinska University Hospital, Stockholm, Sweden; Division of Surgery and Oncology, Department of Clinical Science, Intervention and Technology (CLINTEC), Karolinska Institutet, Stockholm, Sweden; First Propaedeutic Department of Surgery, National and Kapodistrian University of Athens, Hippokration General Hospital, Athens, Greece

**Keywords:** Lauren classification, lymph node metastases, lymphadenectomy

## Abstract

**Background:**

Gastric cancer is a biologically heterogeneous disease with variable patterns of lymph node (LN) metastasis influenced by tumour biology and anatomical location. Contemporary Western data on LN dissemination remain limited.

**Methods:**

This retrospective multicentre study evaluated data from patients with resectable gastric adenocarcinoma who underwent curative-intent gastrectomy at five high-volume European centres. LN retrieval and metastatic involvement were analysed according to tumour location, Lauren histotype, pathological (p) stage, neoadjuvant chemotherapy, and microsatellite status (microsatellite instability (MSI) or microsatellite stable (MSS)). Lymph node stations were analysed individually and grouped into three anatomical tiers based on proximity to the stomach, according to the Japanese Gastric Cancer Association classification.

**Results:**

In all, 950 patients were included in the study. The incidence of LN metastasis in diffuse and mixed histotypes was approximately twice that of intestinal-type tumours, along with higher metastatic rates across multiple perigastric first-tier and selected second-tier stations (*P* < 0.050 to *P* < 0.001). The anatomical pattern of lymphatic spread was site-specific, with consistent drainage pathways across tumour locations. In early gastric cancer, LN metastases were rare and largely confined to stations 3 and 4. Neoadjuvant chemotherapy reduced overall nodal burden without altering the anatomical distribution of metastatic stations. MSI tumours exhibited lower nodal involvement than MSS tumours, with higher rates of node-negative disease (49.3% *versus* 41.3%) and fewer patients with pN3 tumours (15.9% *versus* 27.7%; *P* = 0.044).

**Conclusions:**

Tumour location influenced the anatomical pattern of LN spread, whereas histological and molecular features were associated with differences in metastatic risk and extent. The integration of biological and anatomical factors may support more tailored surgical strategies.

## Teaser

Tumour location determines the anatomical routes of lymph node spread in gastric cancer, whereas histological phenotype and microsatellite status modulate metastatic risk and extent. Diffuse and mixed tumours exhibit a higher nodal burden, whereas MSI cancers show restrained lymphatic dissemination despite advanced local invasion. Integrating biological profiling with anatomically tailored lymphadenectomy may optimize oncological adequacy while avoiding unnecessary nodal dissection.

## Introduction

Surgical resection is considered the cornerstone of curative treatment for gastric cancer. Adequate lymphadenectomy is a crucial component of gastrectomy with curative intent for gastric adenocarcinoma in terms of optimizing survival, increasing staging accuracy, and assessing prognosis^1–3^.

Previous studies have shown that the distribution pattern of lymph node (LN) metastases is associated with the depth of invasion and tumour size, and that the distribution of locoregional LN correlates with the location of the primary tumour^4^. However, this association remains inconsistent because early gastric cancer with submucosal involvement can present with up to 20% nodal metastasis^5,6^, all LN stations may harbour metastases regardless of tumour location, and skip metastasis have been reported^7,8^.

Regarding histotype, previous studies have reported a higher incidence of LN metastasis in diffuse-type tumours than intestinal-type tumours across almost all stations^6^. Yet the exact pattern of lymphatic spread of these subtypes remains unclear. The distribution of LN metastases in microsatellite-instable (MSI) gastric cancer has also been explored, indicating a higher prevalence of node-negative (N0) disease and a more limited involvement of LN stations^9,10^.

However, these observations require further validation in larger prospective cohorts. Insight into the distribution patterns of LN metastases in gastric cancer is essential for the standardization of lymphadenectomy in gastric cancer surgery. Optimizing the extent of lymphadenectomy may enhance surgical radicality and potentially improve overall survival. Therefore, the aim of this study was to assess anatomical LN metastatic patterns of involvement following curative gastrectomy in relation to the three main histotypes (intestinal, diffuse, mixed) and primary tumour location. In particular, the study aimed to distinguish between the anatomical distribution of nodal metastases and metastatic burden. The hypothesis was that tumour location may primarily influence the anatomical distribution of nodal spread, whereas tumour biology, reflected by histotype, may influence the likelihood and extent of LN involvement. Secondary outcomes included lymphatic spread in early gastric cancer and MSI status.

## Methods

### Study design

This study was designed as a retrospective observational cohort study based on prospectively maintained databases and institutional surgical registries. Patients with gastric adenocarcinoma who underwent curative-intent gastrectomy between January 2015 and December 2023 at Karolinska University Hospital, University Hospital of Verona, Amsterdam UMC, and Athens University Hospitals (Laikon University Hospital and Hippokration General Hospital) were eligible for inclusion.

Clinical and histopathological data were compiled in a collaborative database, including patient and tumour characteristics, surgical details, postoperative outcomes, information on LN stations, treatments administered, and follow-up data. When individual LN station data were not available, nodes were grouped into anatomical tiers based on proximity to the stomach, according to the Japanese Gastric Cancer Association classification^2^, to ensure consistency across participating centres and to enable the inclusion of all patients in the analysis. In such cases, LNs were reported in grouped form in the pathological report rather than as individual stations.

For descriptive and analytical purposes, LN stations were categorized into three anatomical tiers: first-tier (perigastric) stations (Nos. 1–7), second-tier stations (Nos. 8–12a), and third-tier (distant) stations (Nos. 12p–16). Although the term ‘tier’ is no longer part of contemporary formal nomenclature, it is used here as a descriptive approach to facilitate anatomical grouping and comparison across centres.

Grouped stations were analysed at the tier level, whereas station-level analyses were restricted to the subset of patients with detailed nodal mapping. Not all nodal stations were explored in every patient, reflecting both the type of surgical procedure performed (for example, station No. 2 is not routinely dissected in distal gastrectomy) and variability in pathological processing, including issues related to specimen handling and potential mixing of nodal stations.

MSI status was primarily determined by immunohistochemical evaluation of mismatch repair proteins (MLH1, MSH2, MSH6, and PMS2). In select patients, results were confirmed by DNA-based microsatellite analysis. MSI data were not available for all patients. This is mainly related to the study period, which includes patients undergoing gastrectomy since 2015, when routine MSI assessment was not yet widely implemented; the use of MSI assessment has progressively increased in more recent years.

The study was approved by the institutional review boards or ethics committees at all participating centres and it was conducted in accordance with national regulations and the Declaration of Helsinki.

### Inclusion criteria

Patients with histologically proven resectable gastric adenocarcinoma and undergoing curative-intent gastrectomy (defined as R0 and R1 resections) with at least D1 lymphadenectomy up to D3 dissection, were included in the study. Patients with R1 resections were included, because all procedures were performed with curative intent (aiming for R0 resection), and microscopic margin involvement was determined only at the final pathological examination. The inclusion of patients with R1 resections was considered appropriate to reflect real-world clinical practice and to avoid selection bias.

### Exclusion criteria

Patients with a diagnosis of gastroesophageal junction Siewert type I–II tumours, R2 resections, recurrent gastric cancer and atypical gastric resections were excluded.

### Statistical analysis

The number and percentage of involved nodes at each station were assessed as a function of Lauren histology. Differences were evaluated using the χ^2^ test or Fisher’s exact test for categorical variables and the Kruskal–Wallis test for continuous variables, because the assumptions of normality and homoscedasticity (variance stability) were not met. Post hoc analysis was performed using Dunn’s test with Bonferroni adjustment.

Determinants of the overall pathological (p) nodal status were evaluated by ordered logistic regression, where nodal status, coded as pN0/pN1/pN2/pN3, was the response variable, Lauren histology was the main determinant, and sex (male/female), age, tumour site, neoadjuvant chemotherapy, pT, and MSI were potential confounders. The proportional odds assumption was assessed and considered acceptable.

A multivariable analysis was also performed using logistic regression, with nodal invasion (absent/present) as the response variable, Lauren histotype as the determinant, and T category, neoadjuvant treatment, type of gastrectomy, extent of lymphadenectomy, and MSI status as potential confounders. Logistic regression was applied to both overall nodal status (pN0/pN+) and grouped nodal stations (first-tier Nos. 1–7 negative *versus* positive; second-tier Nos. 8–12a negative *versus* positive; and distant/third-tier Nos. 12p–16 negative *versus* positive).

Statistical analyses were performed using Stata^®^ versions 16.1 and 19.0 (StataCorp, College Station, TX, USA).

## Results

### Patients and clinical features

The five European high-volume centres contributed a total of 996 consecutive patients, with information on Lauren histotype available for 983. After excluding 33 patients with inadequate staging (28 with < 15 retrieved nodes and 5 with missing information), 950 patients were retained for analysis.

Among these 950 gastric cancer patients, intestinal-type tumours were more frequent (479) than diffuse (346) and mixed-type (125) tumours (*Table 1*). Amsterdam, Laikon, and Hippokration had the highest proportions of intestinal-type tumours (62–67%), whereas Stockholm had the largest proportion of diffuse-type cancers (45%). Verona had a more balanced distribution across intestinal, diffuse, and mixed-type histologies (49%, 38%, and 13%, respectively; *Table S1*). Baseline clinicopathological characteristics are summarized in *Table 1*.

**Table 1:** Main demographic and clinical characteristics of gastric cancer patients according to Lauren histotype

	Intestinal (*n* = 479)	Diffuse (*n* = 346)	Mixed (*n* = 125)	P*
**Sex**				< 0.001
Male	329 (69%)	184 (53%)	78 (62%)	
Female	150 (31%)	162 (47%)	47 (38%)	
Age (years), median (i.q.r.)	72.1 (64.7–78.9)	64.9 (55.4–72.6)	69.7 (60.4–76.9)	< 0.001§
BMI (kg/m^2^), median (i.q.r.)	25.3 (22.8–28.0)	24.7 (22.3–27.8)	25.0 (22.5–28.0)	0.417§
**Site**				< 0.001
Fundus	146 (30%)	41 (12%)	26 (21%)	
Body	121 (25%)	122 (35%)	36 (29%)	
Antrum	203 (42%)	164 (47%)	63 (50%)	
Linitis plastica	9 (2%)	19 (5%)		
**Neoadjuvant chemotherapy**				0.021
No	244 (51%)	151 (44%)	71 (57%)	
Yes	235 (49%)	195 (56%)	54 (43%)	
**Surgical approach**				0.289
Open	294 (61%)	199 (57.5%)	75 (60%)	
Laparoscopy	164 (34%)	121 (35%)	40 (32%)	
Robotic	21 (4%)	26 (7.5%)	10 (8%)	
**Gastrectomy**				0.495
Total	234 (49%)	179 (52%)	57 (46%)	
Distal	240 (50%)	165 (48%)	67 (54%)	
Proximal	5 (1%)	1 (0%)		
**Lymphadenectomy**				0.010 (0.337)
D1	24 (5%)	14 (4%)	6 (5%)	
D1+	73 (15%)	27 (8%)	14 (11%)	
D2	247 (52%)	216 (62%)	79 (63%)	
D2+/D3	134 (28%)	89 (26%)	26 (21%)	
**Retrieved nodes**				0.012§
Median (i.q.r.)	39 (28–51)	42 (30–55)	43 (32–59)	
Mean(s.d.)	42.5(19.9)	44.0(17.7)	47.5(21.4)	

Values are *n* (%) unless otherwise stated. i.q.r., interquartile range; BMI, body mass index; s.d., standard deviation. *χ^2^ test or Fisher’s exact test for categorical variables and §Kruskal–Wallis test for continuous variables with unstable (age) variance.

Sex distribution differed significantly across histotypes (*P* < 0.001): intestinal tumours occurred predominantly in men (69%), whereas diffuse tumours showed a more balanced distribution (53% men, 47% women), and mixed-type tumours had an intermediate pattern (62% men). Patients with diffuse-type gastric cancer were notably younger, with a median age of 64.9 (interquartile range (i.q.r.) 55.4–72.6) years, compared with 72.1 (i.q.r. 64.7–78.9) years for intestinal tumours and 69.7 (i.q.r. 60.4–76.9) years for mixed-type tumours (*P* < 0.001). Body mass index did not differ significantly between groups (*P* = 0.417). Most cancers arose from the antrum, regardless of histotype. However, the second most frequent site of origin was the fundus for the intestinal histotype and the body for the diffuse/mixed histotypes.

Neoadjuvant chemotherapy was more frequently administered to patients with diffuse than intestinal/mixed tumours, whereas surgical approach did not differ among histotypes. Approximately half the patients underwent total gastrectomy, with distal gastrectomy accounting for approximately 48–54% of procedures across groups. Proximal gastrectomy was rare in all subtypes.

D2 dissection was most frequently performed overall, but was particularly common in diffuse (62%) and mixed-type (63%) tumours compared with intestinal tumours (52%). More extended lymphadenectomy (D2+/D3) was performed slightly more often in intestinal cancers (28%) than in diffuse (26%) or mixed-type (21%) cancers. Consequently, the extent of lymphadenectomy differed across histotype (*P* = 0.010). The number of retrieved nodes was slightly higher in mixed and diffuse histotypes than in the intestinal histotype (Dunn’s test, *P* = 0.011 and *P* = 0.054, respectively).

At pathological examination, tumours were more advanced in diffuse and mixed histotypes than in the intestinal histotype (*Table 2*). The incidence of pT4 was 50% and 42% in diffuse and mixed-type tumours respectively, compared with 21% in the intestinal subtype. Conversely, 41% of intestinal tumours were pT0/pT1/pT2, *versus* 28% in diffuse/mixed tumours. Tumour size was not significantly different between histotypes. A similar pattern was observed for pN status: no nodal metastases were recorded in 50% of intestinal tumours, *versus* 36% and 34% of diffuse/mixed tumours, respectively. Conversely, the proportion of pN3 tumours was approximately doubled in diffuse compared with intestinal tumours (35% *versus* 17%). Accordingly, compared with the intestinal histotype, the number of positive nodes was significantly higher in both diffuse and mixed histotypes (*P* < 0.001).

**Table 2:** Main pathological tumour characteristics according to Lauren histotype

	Intestinal (*n* = 479)	Diffuse (*n* = 346)	Mixed (*n* = 125)	P*
**pT**				< 0.001
pT0	27 (5.6%)	12 (3.5%)	4 (3.2%)	
pT1	96 (20.0%)	61 (17.6%)	20 (16.0%)	
pT2	75 (15.7%)	24 (6.9%)	11 (8.8%)	
pT3	179 (37.4%)	76 (22.0%)	38 (30.4%)	
pT4	102 (21.3%)	173 (50.0%)	52 (41.6%)	
**Tumour size (mm)**				0.127§
Median (i.q.r.)	35 (22–52)	40 (25–55)	40 (26–55)	
Mean(s.d.)	39.2(24.1)	46.4(39.6)	45.6(33.3)	
**pN**				< 0.001
pN0	239 (49.9%)	125 (36.2%)	43 (34.4%)	
pN1	84 (17.5%)	41 (11.9%)	20 (16.0%)	
pN2	73 (15.2%)	57 (16.5%)	26 (20.8%)	
pN3	83 (17.3%)	122 (35.4%)	36 (28.8%)	
**Positive nodes**				< 0.001§
Median (i.q.r.)	0 (0–3)	3 (0–11)	2 (0–8)	
Mean(s.d.)	2.9(5.6)	8.1(12.4)	6.3(10.2)	

Values are *n* (%) unless otherwise stated. p, pathological; i.q.r., interquartile range; s.d., standard deviation. *χ^2^ test, except §Kruskal–Wallis test.

### Determinants of overall nodal invasion

In multivariable analysis, pathological nodal status was strongly associated with Lauren histology: the odds of being in a higher pN tier were doubled in diffuse than intestinal histotypes (*Table 3*). As expected, the odds ratio increased exponentially with increasing pT tier. Of note, the presence of MSI as opposed to microsatellite stability (MSS) halved the odds of being in a higher pN tier. Conversely, sex, age, tumour site, and neoadjuvant chemotherapy were not independent predictors of nodal status.

**Table 3:** Multivariable ordered logistic regression analysis for potential demographic and clinical determinants of advanced nodal metastatic disease*

	Odds ratio	*P*
Sex (female *versus* male)	0.76 (0.51, 1.12)	0.164
Age (per 10-year increase)	1.01 (0.85, 1.21)	0.872
**Site**†		
Fundus	1 (Reference)	
Body	0.87 (0.50, 1.50)	0.613
Antrum	1.06 (0.64, 1.76)	0.818
Neoadjuvant CTx (yes *versus* no)	0.84 (0.56, 1.27)	0.403
**pT**		
pT0	1 (Reference)	
pT1	2.16 (0.56, 8.23)	0.261
pT2	4.87 (1.28, 18.57)	0.020
pT3	13.31 (3.73, 47.46)	< 0.001
pT4	29.08 (8.18, 103.4)	< 0.001
MSI (yes *versus* no)	0.54 (0.32, 0.92)	0.022
**Lauren histology**		
Intestinal	1 (Reference)	
Diffuse	2.25 (1.42, 3.58)	0.001
Mixed	1.22 (0.70, 2.10)	0.483

Values in parentheses are 95% confidence intervals. *The analysis was performed on 456 patients with information on MSI status. †Linitis plastica was recoded as tumour arising from the body of the stomach. CTx, chemotherapy; p, pathological; MSI, microsatellite instability.

### Exploration of single nodal stations

**Fig. 1:**
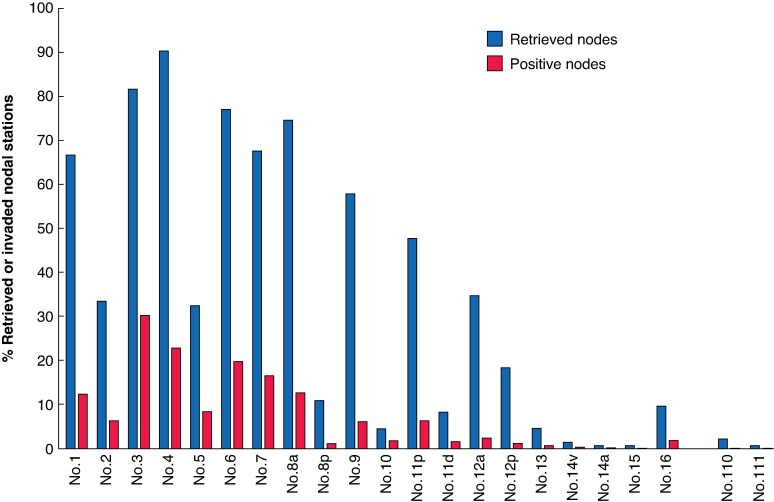
Percentage of lymph nodes retrieved and metastatic involvement by individual nodal station Note, not all nodal stations were explored in every patient. This reflects both the type of surgical procedure performed (for example, No. 2 is less frequently dissected in distal gastrectomy) and variability in pathological processing, including potential mixing of nodal stations.

Information on individual nodal stations was available for 807 of 950 patients (85%) from Stockholm, Amsterdam, and Verona. In this subgroup, the following stations were removed and pathologically examined in more than 70% of patients: No. 3 (81.9% of patients), No. 4 (90.3% of patients), No. 6 (77.1% of patients), and No. 8a (74.6% of patients; *Fig. 1*). In addition, stations No. 1, No. 7, and No. 9 were evaluated in more than half of patients (66.7%, 67.5%, and 58.0%, respectively). Conversely, stations No. 10 (4.3% of patients), No. 13 (4.7% of patients), No. 14 (1.7% of patients), and No. 15 (0.7% of patients), as well as para-aortic (No. 16; 9.7% of patients) and mediastinal (No. 110 (2.1% of patients) and No. 111 (0.7% of patients)) nodes, were rarely explored.

The station with the highest rate of metastasis detection was No. 3, positive in 30.4% of patients, followed by stations No. 4 (22.9%), No. 6 (20.0%), No. 7 (16.6%), No. 8a (12.5%), and No. 1 (12.3%). Stations above No. 8a were positive in no more than 2.5% of patients, apart from stations No. 11p (6.4%) and No. 9 (6.1%).

### LN involvement based on histotype

**Fig. 2:**
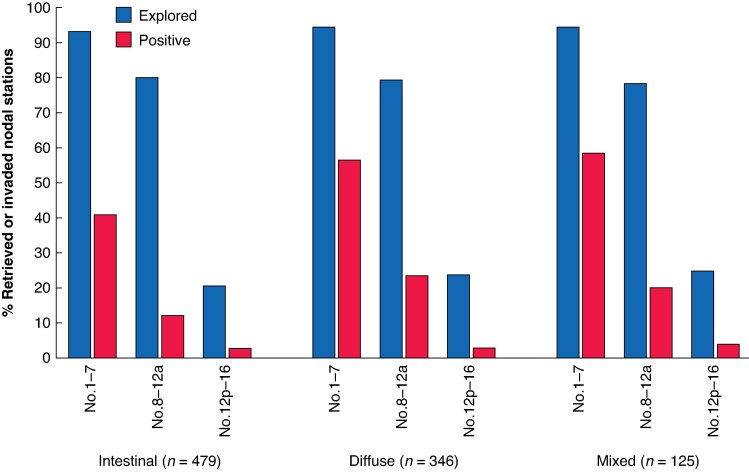
Distribution of retrieved (explored) and metastatic lymph nodes according to anatomical nodal tier and histotype The percentage of lymph nodes retrieved and pathologically examined (explored) and the percentage of metastatic lymph nodes (positive) are shown for each nodal tier. The analysis included 950 patients in total.

Across all histotypes, perigastric LN stations (Nos. 1–7) were explored in nearly all patients (93–95%), whereas second-tier stations (Nos. 8–12a) and distant/third-tier stations (Nos. 12p–16) were retrieved with progressively lower frequency (*Fig. 2*).

Marked differences in nodal positivity emerged among Lauren subtypes (*Fig. S1*). Diffuse and mixed-type tumours presented the greatest metastatic burden: positivity peaked at 57% and 58% across stations Nos. 1–7, respectively, significantly higher than that observed for intestinal tumours (41%; *Fig. 2*). A similar gradient was noted in the case of second-tier nodes, where diffuse and mixed cancers showed substantially greater involvement (23% and 20%, respectively) than intestinal tumours (12%). In contrast, all histotypes showed uniformly low invasion rates in distant stations (Nos. 12p–16), remaining < 5%.

**Fig. 3:**
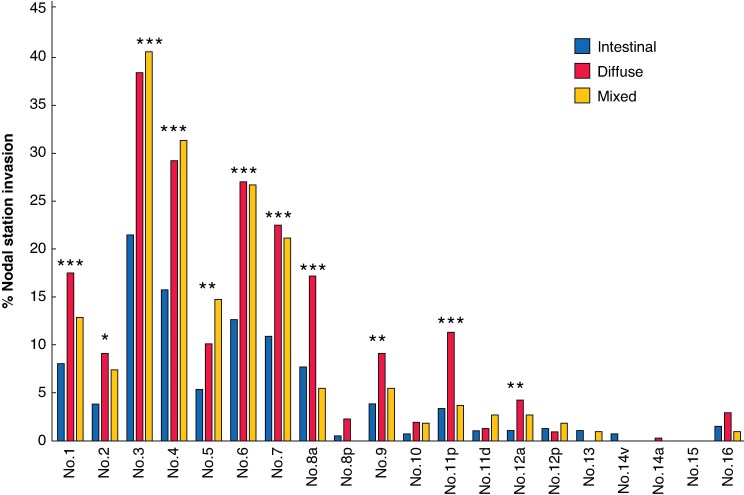
Percentage lymph node station involvement stratified according to Lauren histotype Asterisks indicate statistically significant differences across histotypes (*P < 0.050; **P < 0.010; ***P < 0.001). The analysis included 807 patients in total.

**Fig. 4:**
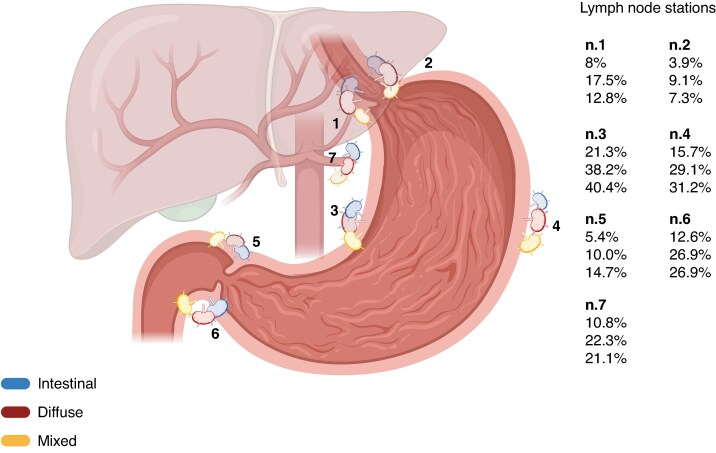
Percentage metastatic involvement for individual lymph node stations within the first-tier (perigastric) nodal group, stratified according to Lauren histotype Lymph node stations are numbered according to the Japanese Gastric Cancer Association classification^2^. The analysis included 807 patients. This figure was created by F Blasa in BioRender.com (https://BioRender.com/avkgm0b).

**Fig. 5:**
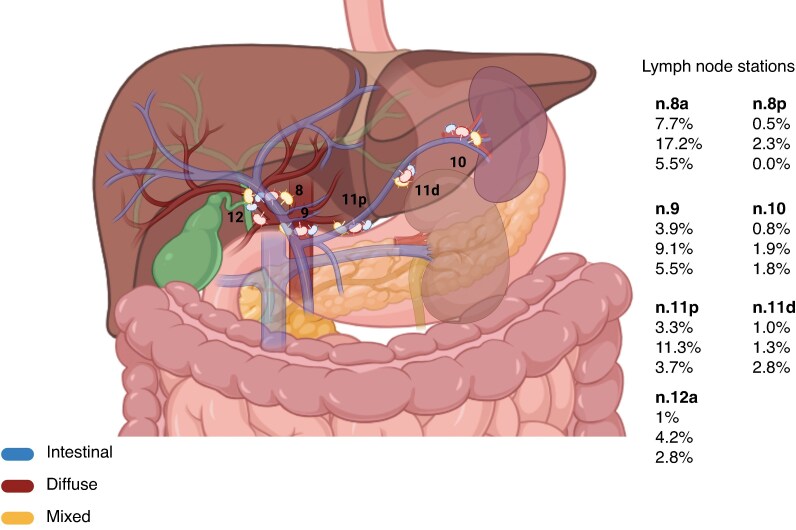
Percentage metastatic involvement of individual lymph node stations within the second-tier nodal group, stratified according to Lauren histotype Lymph node stations are numbered according to the Japanese Gastric Cancer Association classification^2^. The analysis included 807 patients. This figure was created by F Blasa in BioRender.com (https://BioRender.com/avkgm0b).

**Fig. 6:**
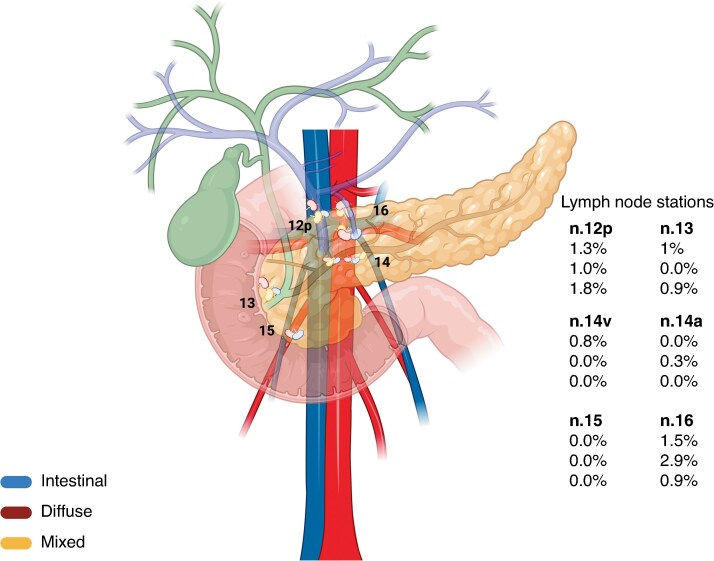
Percentage metastatic involvement of individual lymph node stations within the third-tier nodal group, stratified according to Lauren histotype Lymph node stations are numbered according to the Japanese Gastric Cancer Association classification^2^. The analysis included 807 patients. This figure was created by F Blasa in BioRender.com (https://BioRender.com/avkgm0b).

Station-specific comparisons confirmed this metastatic pattern (*Fig. 3*). Diffuse and mixed cancers exhibited significantly higher positivity than intestinal cancers across all first-tier stations (*Fig. 4*). Significant differences were also detected for stations in the second tier with at least 2% positivity (Nos. 8a, 9, 11p, and 12a; *Fig. 5*); in the latter stations (Nos. 11p and 12a), the proportion of metastatic involvement was approximately doubled in the diffuse than intestinal and mixed histotypes. No significant differences were detected in third-tier stations (*Fig. 6*). A stratified analysis restricted to advanced tumours (pT3–pT4) showed a persistent, although attenuated, higher nodal involvement in diffuse-type tumours compared with the intestinal type, supporting that this association is not solely explained by deeper tumour invasion (*Fig. S2*).

Histotype-specific distribution maps further illustrated these trends. Intestinal-type tumours exhibited a predictable, anatomy-driven pattern, with metastasis concentrated in stations 3 and 4 and only rare involvement beyond D1 nodes. Diffuse-type cancers showed the broadest dissemination, with substantial involvement of multiple perigastric stations and consistent extension into second-tier nodes (Nos. 8a, 9, 10, 11p/11d, and 12a). Mixed-type tumours exhibited an intermediate phenotype: perigastric involvement approached that of diffuse cancers, whereas second-tier spread was less frequent (*Figs S3–S5*).

### LN spread based on tumour site

**Fig. 7:**
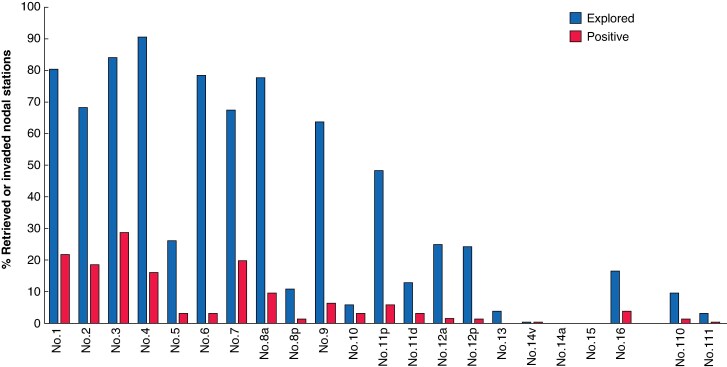
Distribution of retrieved (explored) and metastatic lymph nodes by nodal station in fundus tumours The analysis included 807 patients; fundus tumours were found in 157.

**Fig. 8:**
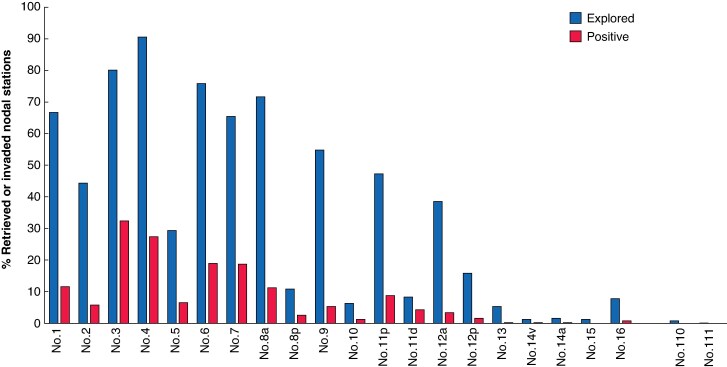
Distribution of retrieved (explored) and metastatic lymph nodes by nodal station in gastric body tumours The analysis included 807 patients; gastric body tumours were found in 241.

**Fig. 9:**
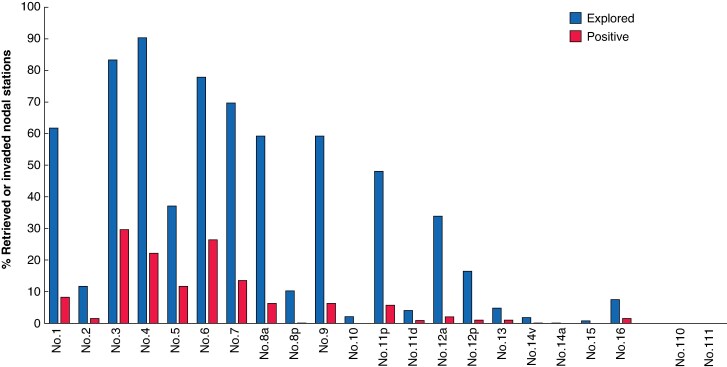
Distribution of retrieved (explored) and metastatic lymph nodes by nodal station in antral tumours The analysis included 807 patients; tumours of the antrum were found in 383.

**Fig. 10:**
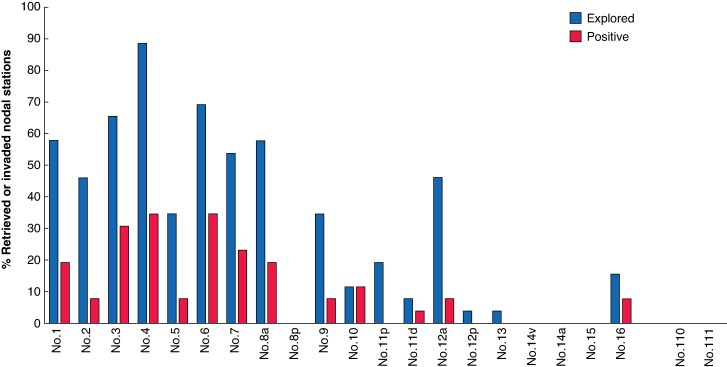
Distribution of retrieved (explored) and metastatic lymph nodes by nodal station in linitis plastica The analysis included 807 patients; linitis plastica was found in 26.

Distinct, site-specific patterns of lymphatic dissemination emerged across gastric tumour locations, reflecting standard D2 dissection pathways. Fundus tumours exhibited a markedly localized metastatic profile, with involvement largely confined to upper perigastric nodes and limited spread to distant stations (*Fig. 7*). Tumours of the gastric body exhibited a broader distribution of perigastric metastases, predominantly in stations 3 and 4, yet retained limited involvement of second-tier nodes (*Fig. 8*). Antral cancers showed a characteristic infrapyloric/suprapyloric pattern, with high positivity in stations 3, 4, and 6 and only sporadic metastases beyond the perigastric field (*Fig. 9*). In contrast, linitis plastica exhibited the most extensive lymphatic spread, involving multiple first- and second-tier stations and occasional distant nodes (*Fig. 10*).

### Individual nodal station involvement in early-upfront gastric cancer

In early gastric cancers treated with upfront surgery (101 patients), LN retrieval remained high across the first-tier stations, particularly stations 3, 4, 6, and 7, where exploration exceeded 70–90% (*Fig. S6*). Despite this extensive retrieval, nodal metastasis was rare. The highest involvement rates were observed in stations 3 and 4, yet even here positivity remained low (∼8–10%). Stations 1, 5, 6, and 7 exhibited only sporadic metastases, each below 5%. Importantly, no meaningful metastatic involvement was detected beyond the perigastric field; positivity in second-tier nodes (Nos. 8a, 9, 11p, 12a) was extremely uncommon, and distant stations (Nos. 12p, 14v, 16) showed virtually no metastasis.

### Individual nodal station involvement after neoadjuvant chemotherapy

Neoadjuvant chemotherapy substantially reduced the overall burden of nodal metastases while preserving the anatomical pattern of spread (*Fig. S7*). The highest involvement proportion remained in stations 3, 4, 6, and 7, suggesting that chemotherapy decreases the extent of nodal involvement rather than shifting the anatomical distribution of metastatic sites. Metastatic involvement beyond the perigastric basin was uncommon, reinforcing that even after systemic therapy, lymphatic dissemination follows predictable pathways. In patients receiving neoadjuvant therapy, the median number of harvested LNs was 40 (i.q.r. 29–53).

### LN exploration and metastatic involvement according to microsatellite status

Several clinicopathological characteristics differed significantly between MSS and MSI tumours. Neoadjuvant chemotherapy was administered more frequently in patients with MSS than MSI (60.3% *versus* 43.5%; *P* = 0.009), reflecting a greater propensity towards preoperative systemic treatment in the case of MSS disease (*Table 4*).

**Table 4:** Neoadjuvant therapy and pathological characteristics according to microsatellite status

	MSS (*n* = 388)	MSI (*n* = 69)	*P*
**Neoadjuvant chemotherapy**	234 (60.3%)	30 (44%)	0.009*
**Lauren’s histology**			< 0.001∗
Intestinal	168 (43.3%)	46 (67%)	
Diffuse	164 (42.3%)	12 (17%)	
Mixed	56 (16.6%)	11 (16%)	
**Depth of tumour invasion**			0.040§
pT0	23 (5.9%)	2 (3%)	
pT1	67 (17.3%)	5 (7%)	
pT2	42 (10.8%)	12 (17%)	
pT3	109 (28.1%)	16 (23%)	
pT4	147 (37.9%)	34 (49%)	
**Nodal invasion**			0.044§
pN0	160 (41.3%)	34 (49%)	
pN1	58 (15.0%)	14 (20%)	
pN2	62 (16.0%)	10 (14%)	
pN3	107 (27.7%)	11 (16%)	

Values are *n* (%). MSS, microsatellite stable; MSI, microsatellite instability; p, pathological. *χ^2^ test for neoadjuvant chemotherapy and Lauren’s histology; §χ^2^ for trend for pT and pN.

Lauren’s classification distribution varied markedly between groups (*P* < 0.001): intestinal type cancers predominated in MSI compared with MSS patients (66.7% *versus* 43.3%, respectively), whereas diffuse histology was substantially less common (17.4% *versus* 42.3%, respectively). MSI tumours also presented with deeper invasion (*P* = 0.040), with nearly half classified as pT4 (compared with 37.9% in the MSS cohort). Despite this greater depth of invasion, MSI cancers exhibited distinct LN metastatic nodal characteristics.

Nodal status (pN category) differed significantly between groups (*P* = 0.044). MSI cancers showed a higher proportion of node-negative disease (pN0: 49.3% *versus* 41.3%) and correspondingly fewer patients with extensive nodal metastasis (pN3: 15.9% *versus* 27.7%).

Despite similar exploration patterns, MSI tumours exhibited markedly lower rates of nodal metastasis across all stations. In the MSS cohort, metastatic involvement was observed in a wide range of stations, including stations 1, 2, 3, 4, 5, 6, 7, 9, 11p, and 12a, reflecting a typical pattern of perigastric and second-tier spread (*Figs S8* and *S9*).

## Discussion

This study provides a detailed characterization of lymphatic dissemination in gastric cancer, suggesting that tumour location is a major determinant of the anatomical pattern of nodal spread.

Diffuse and mixed histotypes had nearly twice the incidence of nodal metastasis compared with intestinal tumours, suggesting an intrinsically lymphotropic behavior^11^, whereas MSI tumours represented a biologically distinct subset with reduced metastatic LN potential. Despite this variability in metastatic burden, the preferential routes of lymphatic spread remained strongly site-specific, with cancers of the fundus, body, and antrum exhibiting highly reproducible, site-specific drainage patterns^12–14^.

Combined, these findings suggest that although tumour biology, here represented by the histological phenotype, predicts how extensively a tumour spreads, tumour location dictates where it spreads^15–17^.

The nodal distribution observed in early gastric cancer (pT0–pT1) confirms the well established low propensity for lymphatic dissemination^18–20^. Despite systematic retrieval of first-tier nodes, metastases were rare and almost exclusively limited to stations 3 and 4, the dominant drainage pathways of the lesser curvature. The absence of meaningful involvement of distant stations underscores the limited biological capacity of early tumours to penetrate deeper lymphatic channels^21,22^. These findings support the oncological safety of conservative surgical strategies in appropriately selected patients. Furthermore, the low incidence of metastasis beyond the perigastric field suggests that routine D2 lymphadenectomy may not be necessary for accurately staged early tumours and that D1/D1+ lymphadenectomy could also be sufficient in Western patient populations, in accordance with Japanese^2,23^ and Korean guidelines. However, these results should be interpreted with caution given the limited sample size, the retrospective nature of staging, and potential differences between Western and Eastern populations, and should be considered hypothesis-generating.

These observations also offer important insights into how neoadjuvant chemotherapy modifies LN metastatic propensity. Although systemic therapy substantially reduced the overall burden of nodal metastases, the underlying anatomical pattern of spread remained remarkably stable^23^. Among advanced tumours (pT2–pT4), the distribution of metastatic stations differed only marginally between patients treated upfront and those receiving neoadjuvant chemotherapy (*Fig. S10*). The hierarchical involvement of stations 3, 4, 6, and 7 was preserved in both groups, with only modest decreases in positivity observed after chemotherapy.

Overall, neoadjuvant treatment appears to reduce nodal tumour burden without altering the fundamental lymphatic dissemination pathways. Consequently, even in pretreated patients, a systematic D2 lymphadenectomy remains essential to ensure complete regional clearance and accurate pathological staging. This reinforces current guideline recommendations and highlights the biological consistency of lymphatic spread, regardless of treatment timing.

The markedly reduced nodal involvement observed in MSI tumours, despite comparable lymphadenectomy (*Figs S11* and *S12*), supports the concept that MSI gastric cancer represents a biologically less aggressive entity with limited propensity for lymphovascular dissemination^24,25^.

However, the relatively small number of MSI patients in this cohort may limit the statistical power of subgroup analyses, and these findings should therefore be interpreted with caution.

This raises the possibility that MSI status may eventually guide selective de-escalation of nodal dissection, although prospective evidence is required before altering current surgical standards. Importantly, even with a higher proportion of pT4 lesions, MSI tumours remained substantially less lymphotropic: and only a minority exhibited high nodal burden compared with MSS cancers. This decoupling of tumour depth from nodal spread is a hallmark of MSI biology and is consistent with the patterns demonstrated in both the station-specific and overall nodal-count analyses in the present study. Together, these findings underscore that MSI gastric cancers combine features of advanced local invasion with restrained lymphatic dissemination, a biological paradox that carries important implications for surgical extent, prognostic assessment, and treatment selection.

This study has several notable strengths. It represents one of the largest Western multicentre cohorts to date with detailed, station-level LN mapping, allowing robust comparisons across tumour location, histotype, and MSI status. The inclusion of high-volume European centres with standardized D2 surgery and pathological assessment enhances the generalizability of the findings. Moreover, the combined analysis of clinical, pathological, and treatment variables offers a comprehensive evaluation of factors influencing nodal spread. Nonetheless, some limitations should be acknowledged. The retrospective design introduces the possibility of selection bias and unmeasured confounding. Although meticulous efforts were made to harmonize data across centres, some heterogeneity in pathological processing and station retrieval is unavoidable. Station-level analyses were restricted to centres providing detailed nodal station reporting, which reduced the sample size for these analyses and may have limited statistical power for less frequently retrieved stations. Moreover, some nodal stations were retrieved in only a limited number of patients. It should be noted that posterior stations and other less frequently retrieved stations, such as station No. 10, are not routinely dissected as part of standard lymphadenectomy but are typically retrieved only when there is a high suspicion of involvement. For this reason, nodal positivity rates were calculated only among explored stations, which may introduce detection bias and potentially lead to underestimation of involvement in rarely dissected stations. In addition, MSI status was not uniformly available, reducing the sample size for subgroup analyses. Finally, this study was not designed to assess long-term oncological outcomes or survival, which should be addressed in future prospective studies, such as the TIGER study^26^.

In summary, in this large, multi-institutional study, patterns of lymphatic spread in gastric cancer were associated with both biological characteristics and tumour location. Tumour location appeared to be a major determinant of the anatomical distribution of nodal metastases, whereas histological subtype and microsatellite status were associated with differences in metastatic burden. These findings underscore the importance of integrating histological and molecular profiling with anatomically tailored lymphadenectomy. Further prospective studies are needed to validate these findings and further define their clinical implications.

## Supplementary Material

zrag076_Supplementary_Data

## Data Availability

The data that support the findings of this study are available from the corresponding author upon reasonable request.

## References

[1] Di Leo A, Marrelli D, Roviello F, Bernini M, Minicozzi AM, Giacopuzzi S, et al Lymph node involvement in gastric cancer for different tumour sites and T stage. J Gastrointest Surg 2007;11:1146–1153 17576611 10.1007/s11605-006-0062-2

[2] Japanese Gastric Cancer Association . Japanese gastric cancer treatment guidelines 2021 (6th edition). Gastric Cancer 2023;26:1–25 36342574 10.1007/s10120-022-01331-8 PMC9813208

[3] Lordick F, Carneiro F, Cascinu S, Fleitas T, Haustermans K, Piessen G, et al Gastric cancer: ESMO clinical practice guideline for diagnosis, treatment and follow-up. Ann Oncol 2022;33:1005–1020 35914639 10.1016/j.annonc.2022.07.004

[4] Brisinda G, Chiarello MM, Fico V, Puccioni C, Crocco A, Bianchi V, et al Pattern of distribution of lymph node metastases in individual stations in middle and lower gastric carcinoma. Cancers (Basel) 2023;15:2139 37046800 10.3390/cancers15072139 PMC10093249

[5] Park DJ, Lee HK, Lee HJ, Lee HS, Kim WH, Yang HK et al Lymph node metastasis in early gastric cancer with submucosal invasion: feasibility of minimally invasive surgery. World J Gastroenterol 2004;10:3549–3552 15534904 10.3748/wjg.v10.i24.3549 PMC4611990

[6] de Jongh C, Triemstra L, van der Veen A, Brosens LAA, Luyer MDP, Stoot JHMB et al Pattern of lymph node metastases in gastric cancer: a side-study of the multicenter LOGICA trial. Gastric Cancer 2022;25:1060–1072 36103060 10.1007/s10120-022-01329-2 PMC9587950

[7] Polom K, Marrelli D, Pascale V, Ferrara F, Voglino C, Marini M et al The pattern of lymph node metastases in microsatellite unstable gastric cancer. Eur J Surg Oncol 2017;43:2341–2348 28942235 10.1016/j.ejso.2017.09.007

[8] Choi YY, An JY, Guner A, Kang DR, Cho I, Kwon IG et al Skip lymph node metastasis in gastric cancer: is it skipping or skipped? Gastric Cancer 2016;19:206–215 25708370 10.1007/s10120-015-0472-5

[9] Lo KH, Huang KH, Fang WL, Lin SC, Hung YP, Chen MH et al Characteristics and prognosis of skip lymph node metastasis in gastric cancer: a retrospective study. World J Surg Oncol 2025;23:301 40707976 10.1186/s12957-025-03951-7 PMC12291338

[10] Cancer Genome Atlas Research Network . Comprehensive molecular characterization of gastric adenocarcinoma. Nature 2014;513:202–209 25079317 10.1038/nature13480 PMC4170219

[11] Kajitani T . The general rules for the gastric cancer study in surgery and pathology. Part I: clinical classification. Jpn J Surg 1981;11:127–139 7300058 10.1007/BF02468883

[12] Akagi T, Shiraishi N, Kitano S. Lymph node metastasis of gastric cancer. Cancers (Basel) 2011;3:2141–2159 24212800 10.3390/cancers3022141 PMC3757408

[13] Maruyama K, Gunvén P, Okabayashi K, Sasako M, Kinoshita T. Lymph node metastases of gastric cancer: general pattern in 1931 patients. Ann Surg 1989;210:596–602 2818028 10.1097/00000658-198911000-00005 PMC1357792

[14] Lirosi MC, Biondi A, Ricci R. Surgical anatomy of gastric lymphatic drainage. Transl Gastroenterol Hepatol 2017;2:14 28447049 10.21037/tgh.2016.12.06 PMC5388628

[15] van der Woude CJ, Kleibeuker JH, Tiebosch AT, Homan M, Beuving A, Jansen PLM et al Diffuse and intestinal type gastric carcinomas differ in their expression of apoptosis-related proteins. J Clin Pathol 2003;56:699–702 12944556 10.1136/jcp.56.9.699 PMC1770046

[16] Ikoma N, Agnes A, Chen HC, Wang X, Blum MM, Das P et al Linitis plastica: a distinct type of gastric cancer. J Gastrointest Surg 2020;24:1018–1025 31754987 10.1007/s11605-019-04422-7

[17] Kunisaki C, Shimada H, Nomura M, Matsuda G, Otsuka Y, Ono H et al Distribution of lymph node metastasis in gastric carcinoma. Hepatogastroenterology 2006;53:468–472 16795994

[18] Hu X, Cao L, Yu Y. [Lymph node metastasis and prognosis of gastric cancer without serosal invasion.] Zhonghua Wei Chang Wai Ke Za Zhi 2012;15:133–136 [in Chinese] 22368018

[19] Zhang CD, Ning FL, Zeng XT, Dai DQ. Lymphovascular invasion as a predictor for lymph node metastasis and a prognostic factor in gastric cancer patients under 70 years of age. Int J Surg 2018;53:214–220 29609047 10.1016/j.ijsu.2018.03.073

[20] Park JW, Ahn S, Lee H, Min BH, Lee JH, Rhee PL et al Predictive factors for lymph node metastasis in early gastric cancer with lymphatic invasion after endoscopic resection. Surg Endosc 2017;31:4419–4424 28378075 10.1007/s00464-017-5490-4

[21] Shannon AB, Straker RJ 3rd, Keele L, Fraker DL, Roses RE, Miura JT et al Lymph node evaluation after neoadjuvant chemotherapy for patients with gastric cancer. Ann Surg Oncol 2022;29:1242–1253 34601642 10.1245/s10434-021-10803-7

[22] Ratti M, Lampis A, Hahne JC, Passalacqua R, Valeri N et al Microsatellite instability in gastric cancer: molecular bases, clinical perspectives, and new treatment approaches. Cell Mol Life Sci 2018;75:4151–4162 30173350 10.1007/s00018-018-2906-9 PMC6182336

[23] Kim TH, Park SR, Ryu KW, Choi M, Kim B-H, Eom BW et al Korean practice guidelines for gastric cancer 2022: an evidence-based, multidisciplinary approach. J Gastric Cancer 2023;23:e13 10.5230/jgc.2023.23.e11 PMC9911619 36750993

[24] Cristescu R, Lee J, Nebozhyn M, Kim KM, Ting JC, Wong SS et al Molecular analysis of gastric cancer identifies subtypes associated with distinct clinical outcomes. Nat Med 2015;21:449–456 25894828 10.1038/nm.3850

[25] Tan IB, Ivanova T, Lim KH, Ong CW, Deng N, Lee J et al Intrinsic subtypes of gastric cancer based on gene expression patterns predict survival and respond differently to chemotherapy. Gastroenterology 2011;141:476–485 21684283 10.1053/j.gastro.2011.04.042 PMC3152688

[26] Hagens ERC, van Berge Henegouwen MI, van Sandick JW, Cuesta MA, van der Peet DL, Heisterkamp J et al Distribution of lymph node metastases in esophageal carcinoma (TIGER study): study protocol of a multinational observational study. BMC Cancer 2019;19:662 31272485 10.1186/s12885-019-5761-7 PMC6610993

