# Supplementary material for: Pattern of lymph node spread in gastric cancer: Western multicenter retrospective study
Source: BJS Open. 2026 Jul 3;10(4):zrag076. doi: 10.1093/bjsopen/zrag076 (PMC13330934; doi:10.1093/bjsopen/zrag076)
Supplement: zrag076_Supplementary_Data [file zrag076_supplementary_data.zip › Supplementary_material_MS.docx]

**Pattern of lymph node spread in gastric cancer: A Western multicenter retrospective study**

Francesca Blasa^1^, Giuseppe Verlato^2^, Hidde Overtoom^3,4^, Martina Hermez Chole^5,6^, Fabrizio Tedone^1^, Federica Filippini^1^, [Markos Despotidis](https://pubmed.ncbi.nlm.nih.gov/?term=%22Despotidis%20M%22%5BAuthor%5D)^7^, Evgenia Mela^8^, Tania Triantafyllou^8^, Dimitrios Schizas^7^,  [Dimitrios Theodorou](https://pubmed.ncbi.nlm.nih.gov/?term=%22Theodorou%20D%22%5BAuthor%5D)^8^, Magnus Nilsson^5,6^, Suzanne S. Gisbertz^3,4^, Maria Bencivenga^1^

Ioannis Rouvelas^5,6,8^

1. General and Upper GI Surgery Division, Department of Surgery, University of Verona, Verona, Italy.
2. Department of Diagnostics and Public Health University of Verona Verona, Italy.
3. Department of Surgery, Amsterdam UMC location University of Amsterdam, Amsterdam, the Netherlands.
4. Cancer Treatment and Quality of Life, Cancer Center Amsterdam - Amsterdam, the Netherlands.
5. Department of Upper Abdominal Surgery, Center for Digestive Diseases, Karolinska University Hospital, Huddinge, Stockholm, Sweden.
6. Division of Surgery and Oncology, Department of Clinical Science, Intervention and Technology (CLINTEC), Karolinska Institutet, Stockholm, Sweden.
7. First Department of Surgery, National and Kapodistrian University of Athens, Laikon General Hospital, Athens, Greece.
8. First Propaedeutic Department of Surgery, National and Kapodistrian University of Athens, Hippocration General Hospital, Athens, Greece.

**Corresponding author**: Professor Ioannis Rouvelas, Department of Upper Abdominal Surgery, Center for Digestive Diseases, Karolinska University Hospital, Huddinge, Stockholm, Sweden. Email: Ioannis.rouvelas@ki.se

**Supplementary Materials - Index**

| **Supplementary Figures and Tables** |  |
| --- | --- |
| Supplementary figure 1. | *pag. 3* |
| Supplementary figure 2. | *pag. 4* |
| Supplementary figure 3. | *pag. 5* |
| Supplementary figure 4. | *pag. 6* |
| Supplementary figure 5. | *pag. 7* |
| Supplementary figure 6. | *pag. 8* |
| Supplementary figure 7. | *pag. 9* |
| Supplementary figure 8. | *pag. 10* |
| Supplementary figure 9. | *pag. 11* |
| Supplementary figure 10. | *pag. 12* |
| Supplementary figure 11. | *pag. 13* |
| Supplementary figure 12. | *pag. 14* |
| Supplementary Table 1 | *pag. 15* |

**Supplementary Figures and Tables**

**Supplementary figure 1.**

Distribution of the number of metastatic lymph nodes stratified by Lauren histology, shown as box-and-whisker plots.

**
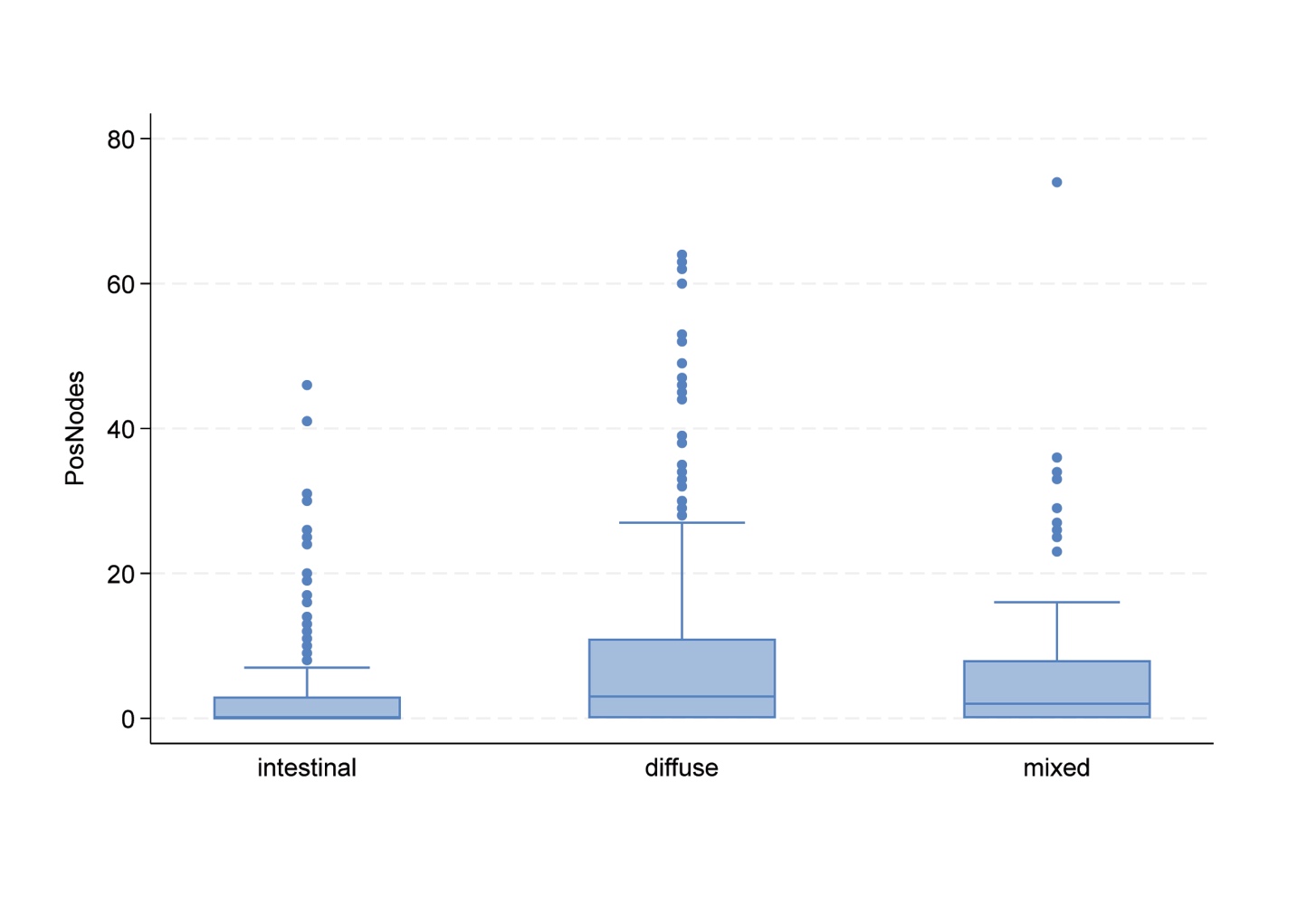
**

**Supplementary figure 2.**

Percentage of metastatic involvement of individual lymph node stations according to Lauren histotype in advanced gastric cancer. (A) pT3 tumours; (B) pT4 tumours. Bars represent intestinal-type (blue), diffuse-type (red), and mixed-type (yellow) gastric cancer. Asterisks indicate statistically significant differences between histotypes (*P < 0.05, **P < 0.01, ***P < 0.001).

**
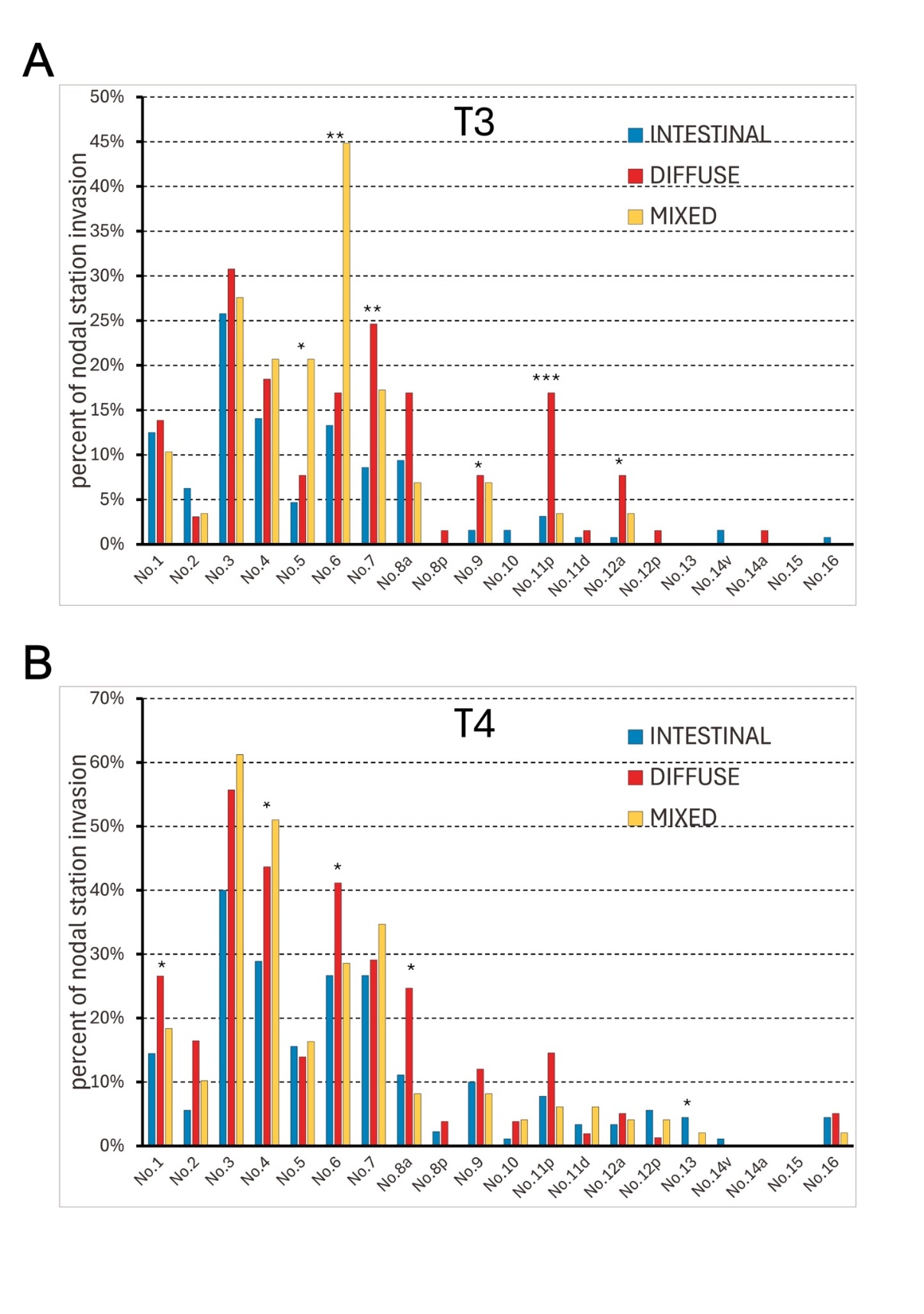
**

**Supplementary figure 3**


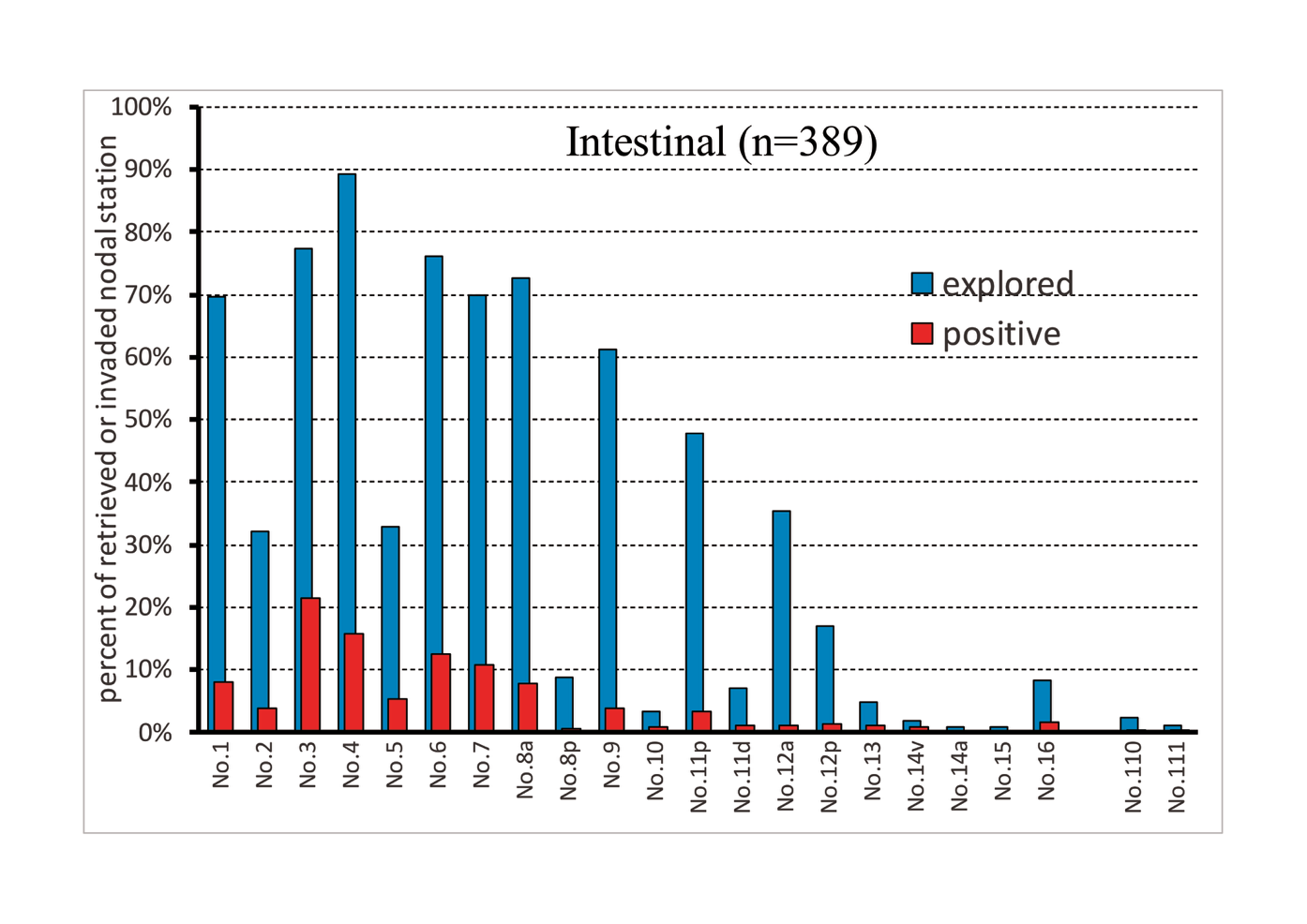
Distribution of retrieved and metastatic lymph nodes across individual nodal stations in intestinal-type gastric cancer. Blue bars represent retrieved lymph nodes and red bars metastatic lymph nodes. A total of 389 patients were included.

**Supplementary figure 4**.


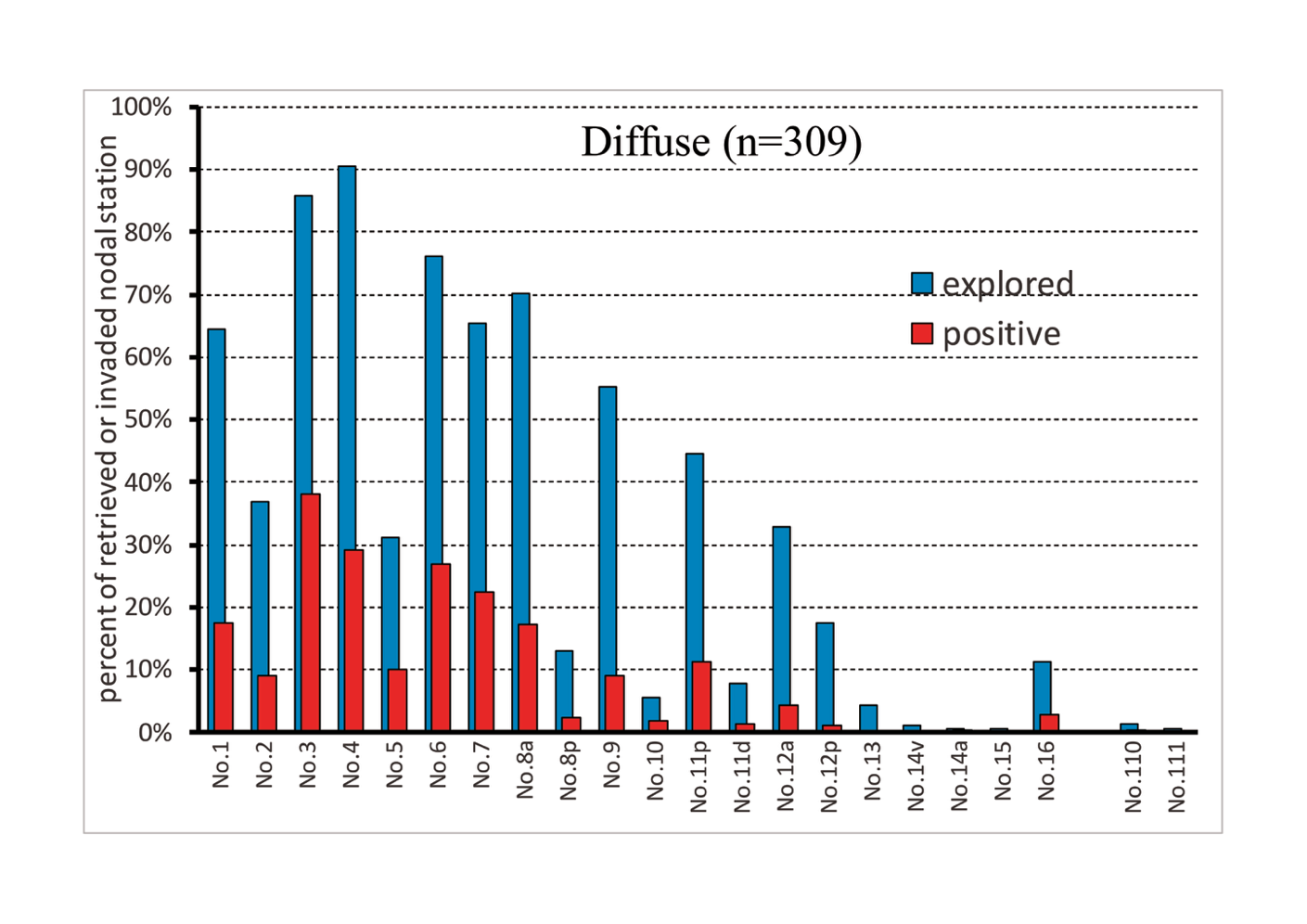
Percentage of retrieved and metastatic lymph nodes by individual nodal station in diffuse-type gastric cancer. Blue bars indicate the proportion of lymph nodes retrieved and pathologically examined, whereas red bars represent the proportion of metastatic lymph nodes. A total of 309 patients were included.

**Supplementary figure 5**.

**
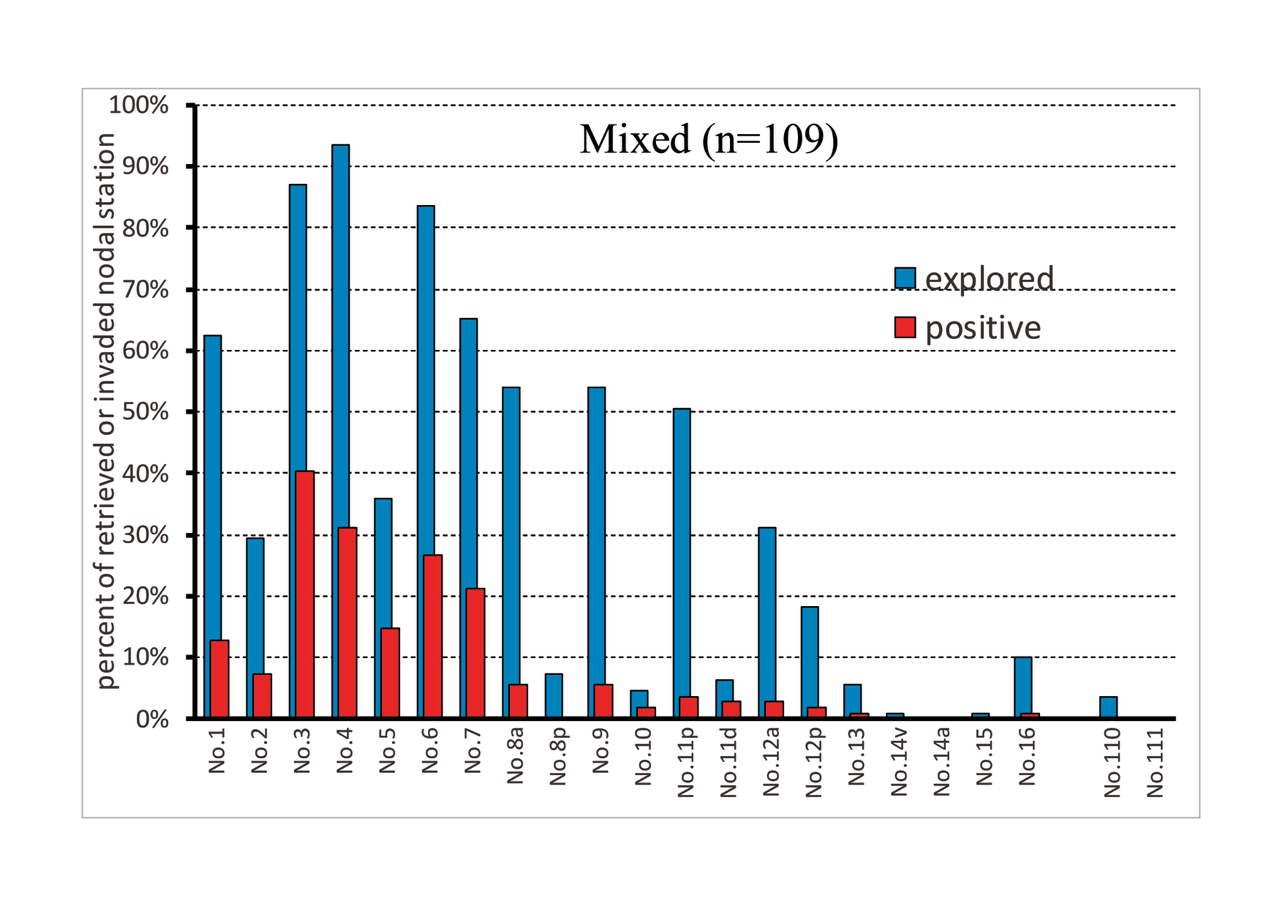
**Percentage of retrieved and metastatic lymph nodes by individual nodal station in mixed-type gastric cancer. Blue bars indicate the proportion of lymph nodes retrieved and pathologically examined, whereas red bars represent the proportion of metastatic lymph nodes. A total of 109 patients were included.

**Supplementary figure 6.**

**
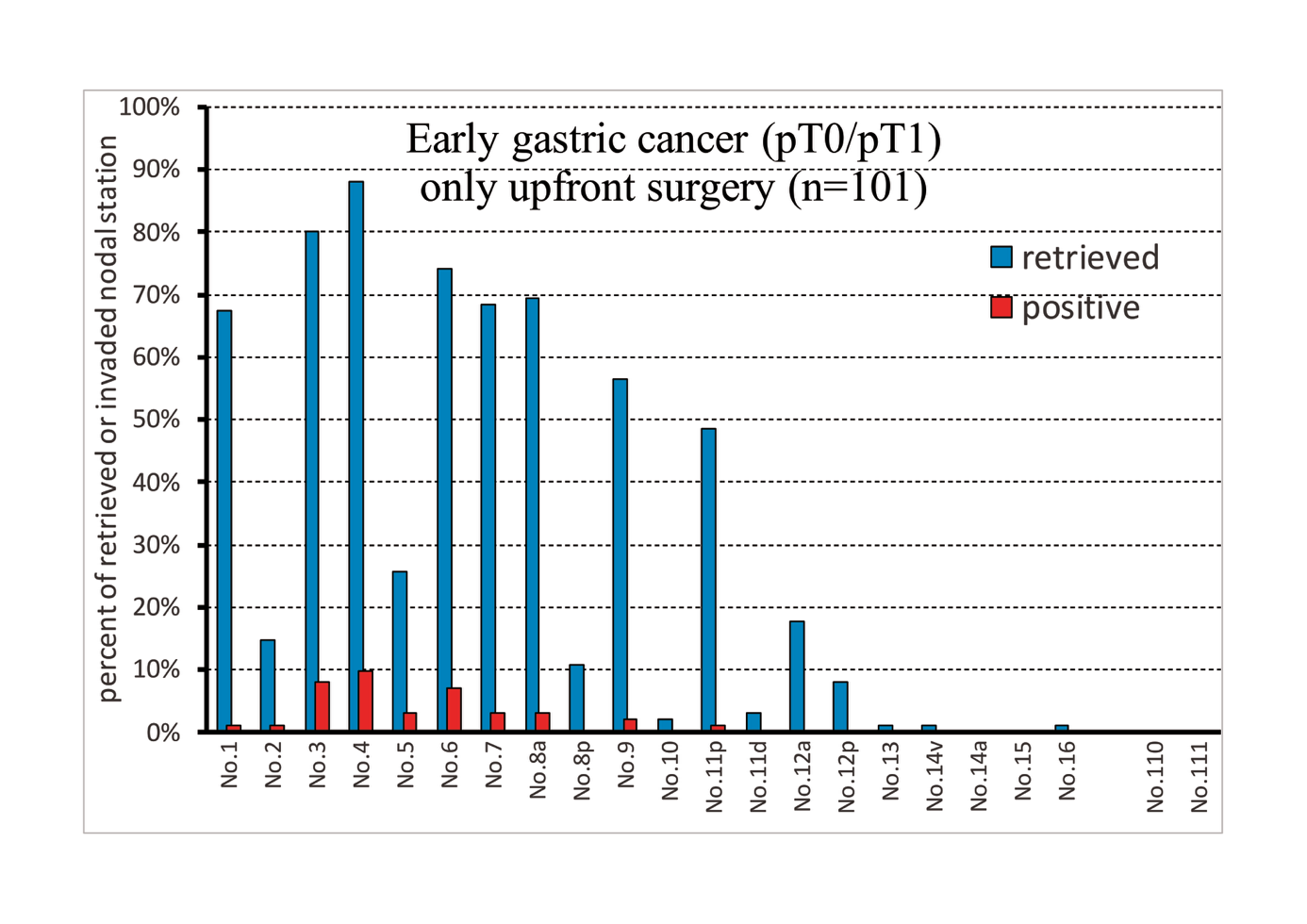
**Percentage of retrieved and metastatic lymph nodes by individual nodal station in patients with early gastric cancer (pT0/pT1) treated with upfront surgery. Blue bars indicate the proportion of lymph nodes retrieved and pathologically examined, whereas red bars represent the proportion of metastatic lymph nodes. The analysis includes 101 patients.

**Supplementary figure 7.**

Percentage of retrieved and metastatic lymph nodes by individual nodal station in patients treated with neoadjuvant chemotherapy. Blue bars indicate the proportion of lymph nodes retrieved and pathologically examined, whereas red bars represent the proportion of metastatic lymph nodes.

**
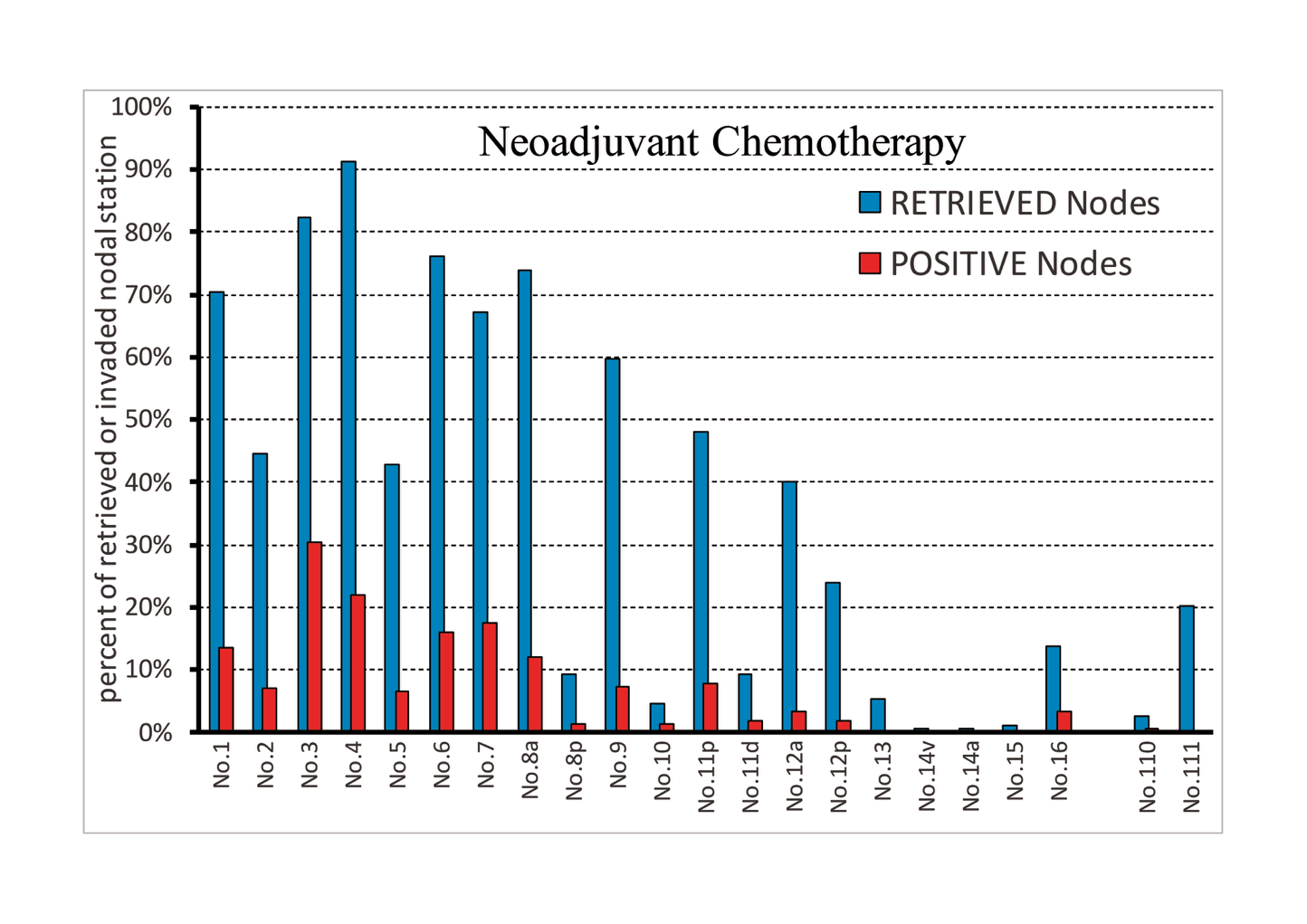
**

**Supplementary figure 8.**

Percentage of retrieved and metastatic lymph nodes by individual nodal station in microsatellite stable (MSS) tumours. Blue bars indicate the proportion of lymph nodes retrieved and pathologically examined, whereas red bars represent the proportion of metastatic lymph nodes. **
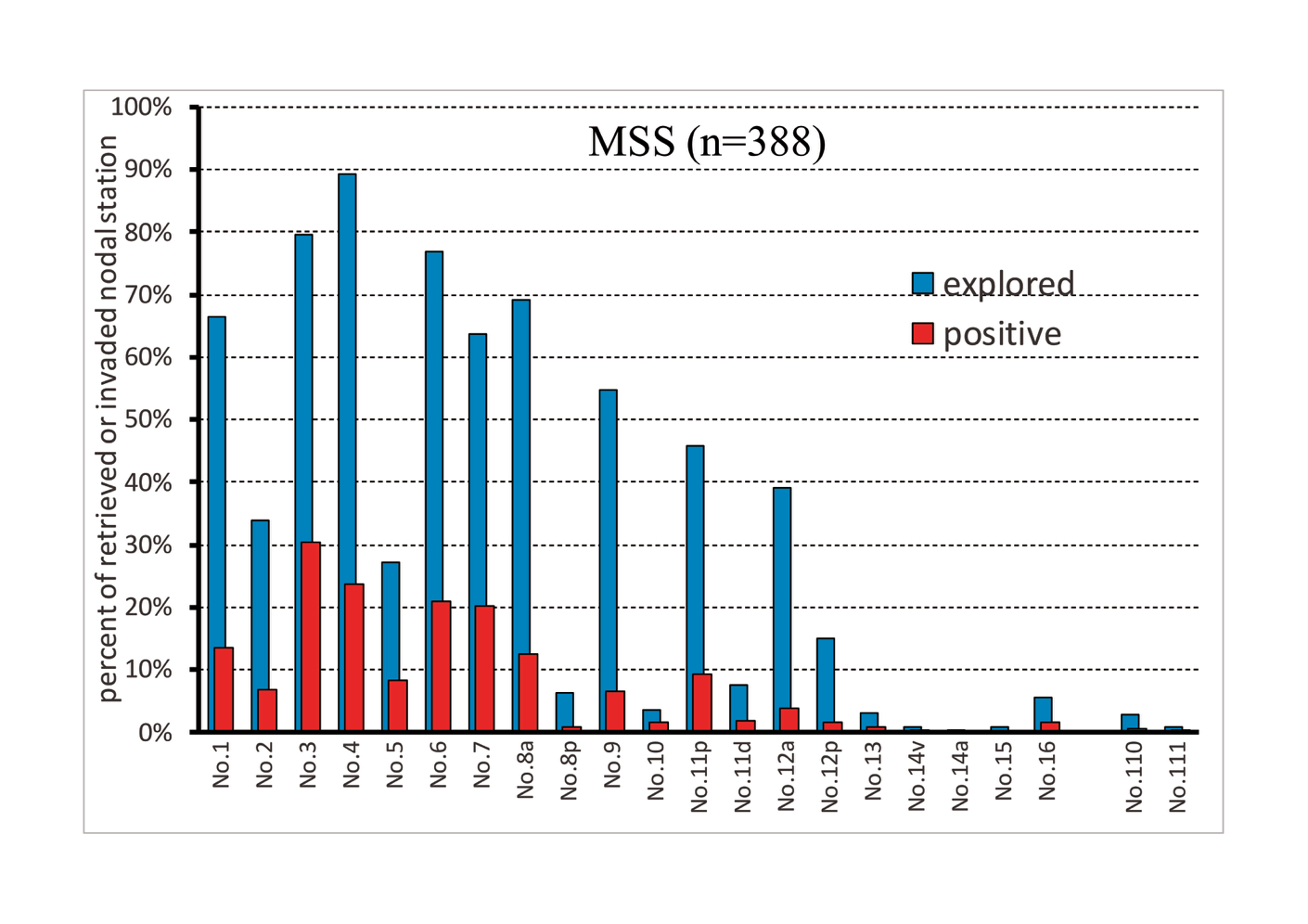
**

**Supplementary figure 9.**

**
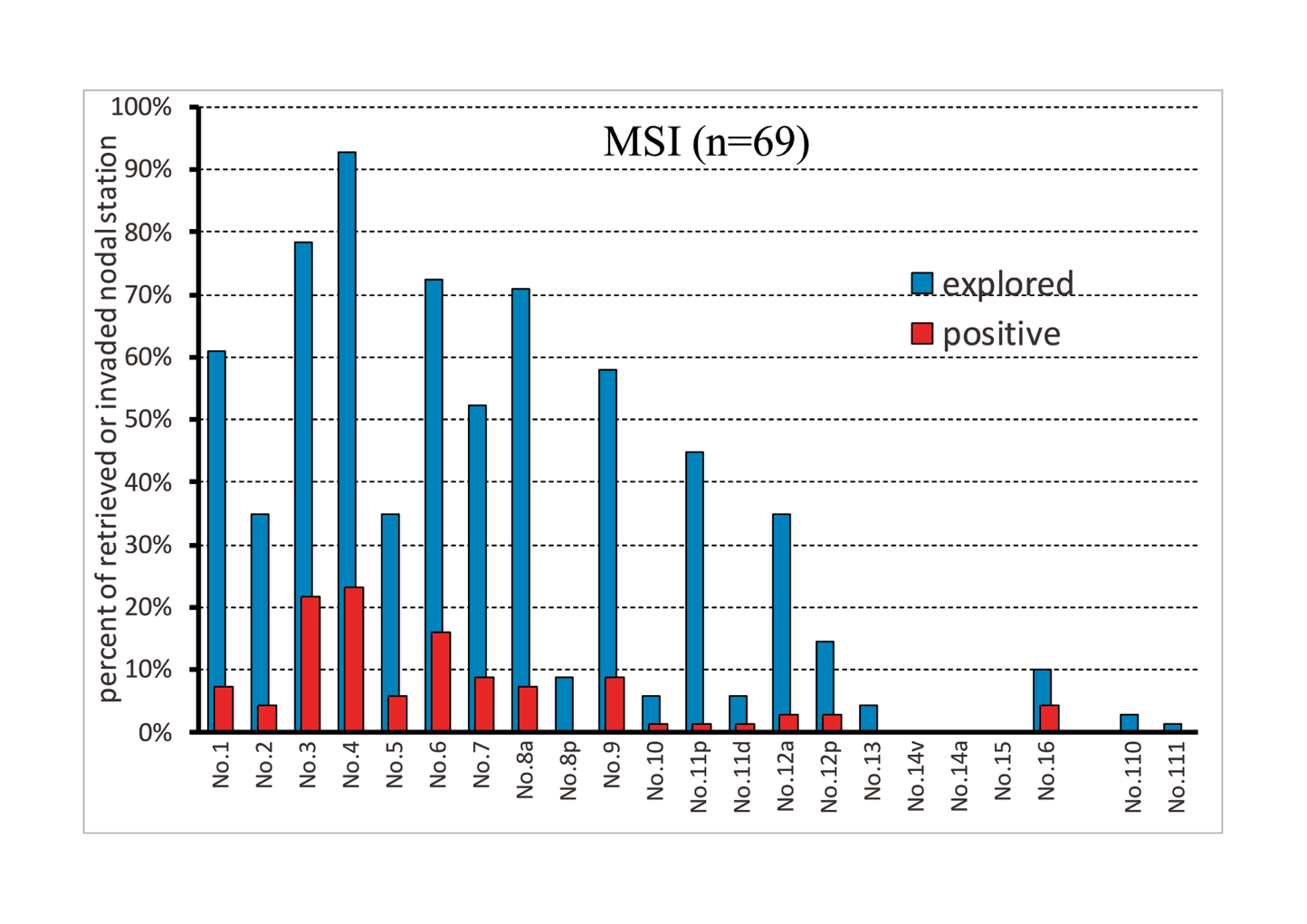
**Percentage of retrieved and metastatic lymph nodes by individual nodal station in microsatellite instability (MSI) tumours. Blue bars indicate the proportion of lymph nodes retrieved and pathologically examined, whereas red bars represent the proportion of metastatic lymph nodes.

**Supplementary figure 10.**

**
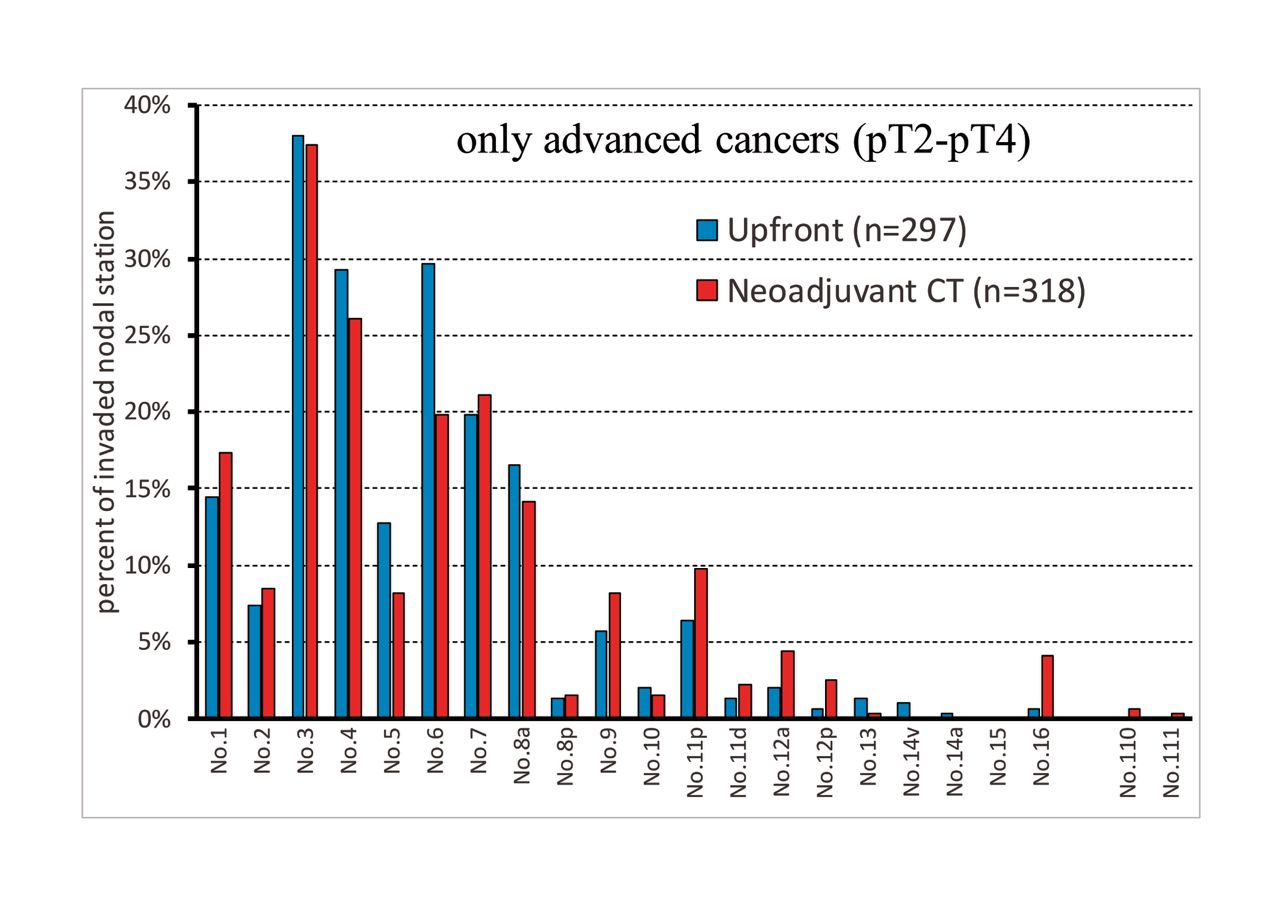
**Percentage of metastatic lymph node involvement by individual nodal station in patients with advanced gastric cancer (pT2–pT4) according to treatment strategy. Blue bars represent patients treated with upfront surgery (n = 297), whereas red bars indicate patients treated with neoadjuvant chemotherapy followed by surgery (n = 318).

**Supplementary figure 11.**

**
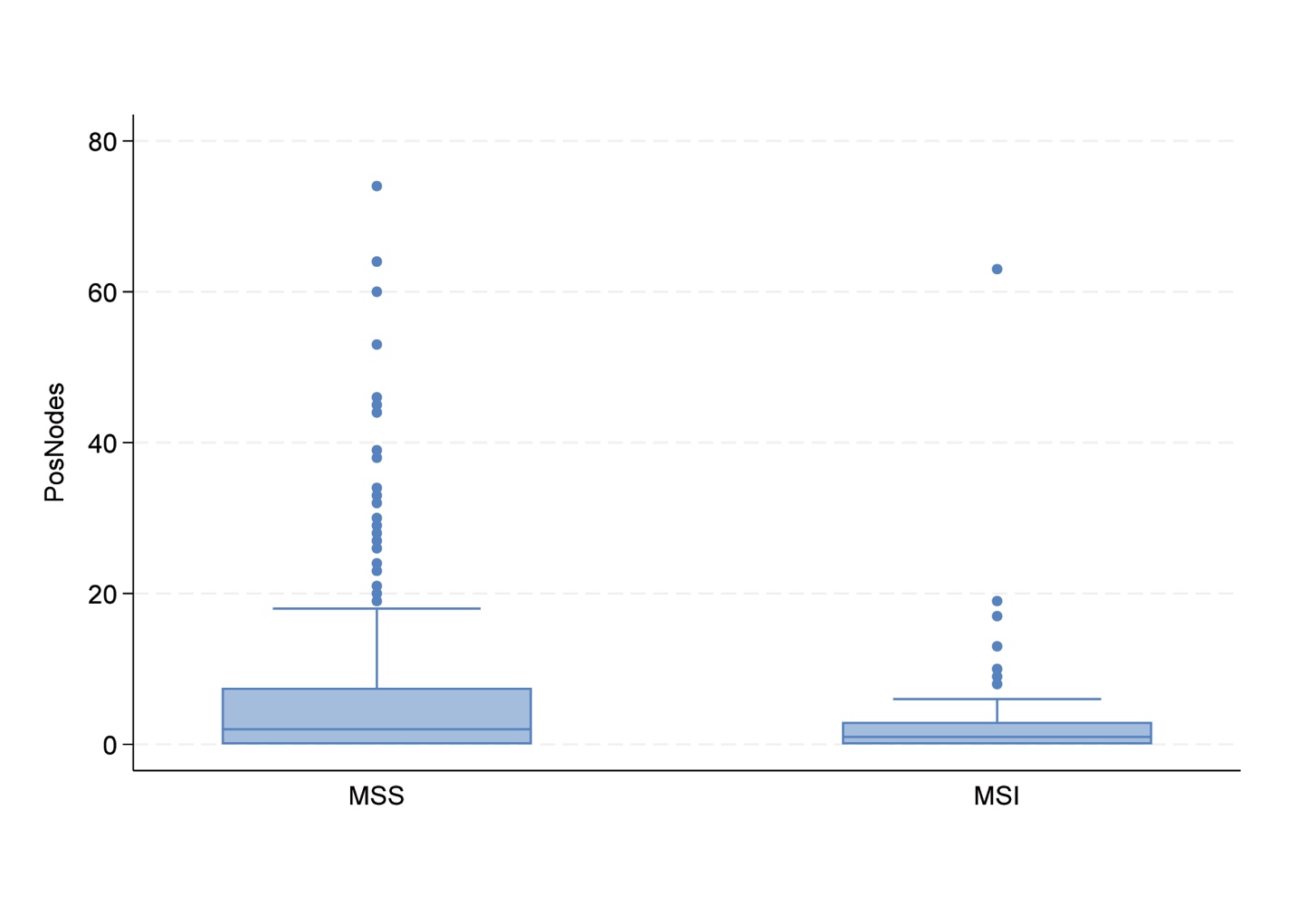
**Distribution of the number of metastatic lymph nodes stratified by microsatellite status, shown as box-and-whisker plots.

**Supplementary figure 12.**

Percentage of retrieved and metastatic lymph nodes by anatomical nodal tier according to microsatellite status. Blue bars indicate the proportion of lymph nodes retrieved and pathologically examined, whereas red bars represent the proportion of metastatic lymph nodes. MSS tumours (n = 388) are shown on the left and MSI tumours (n = 69) on the right.

**
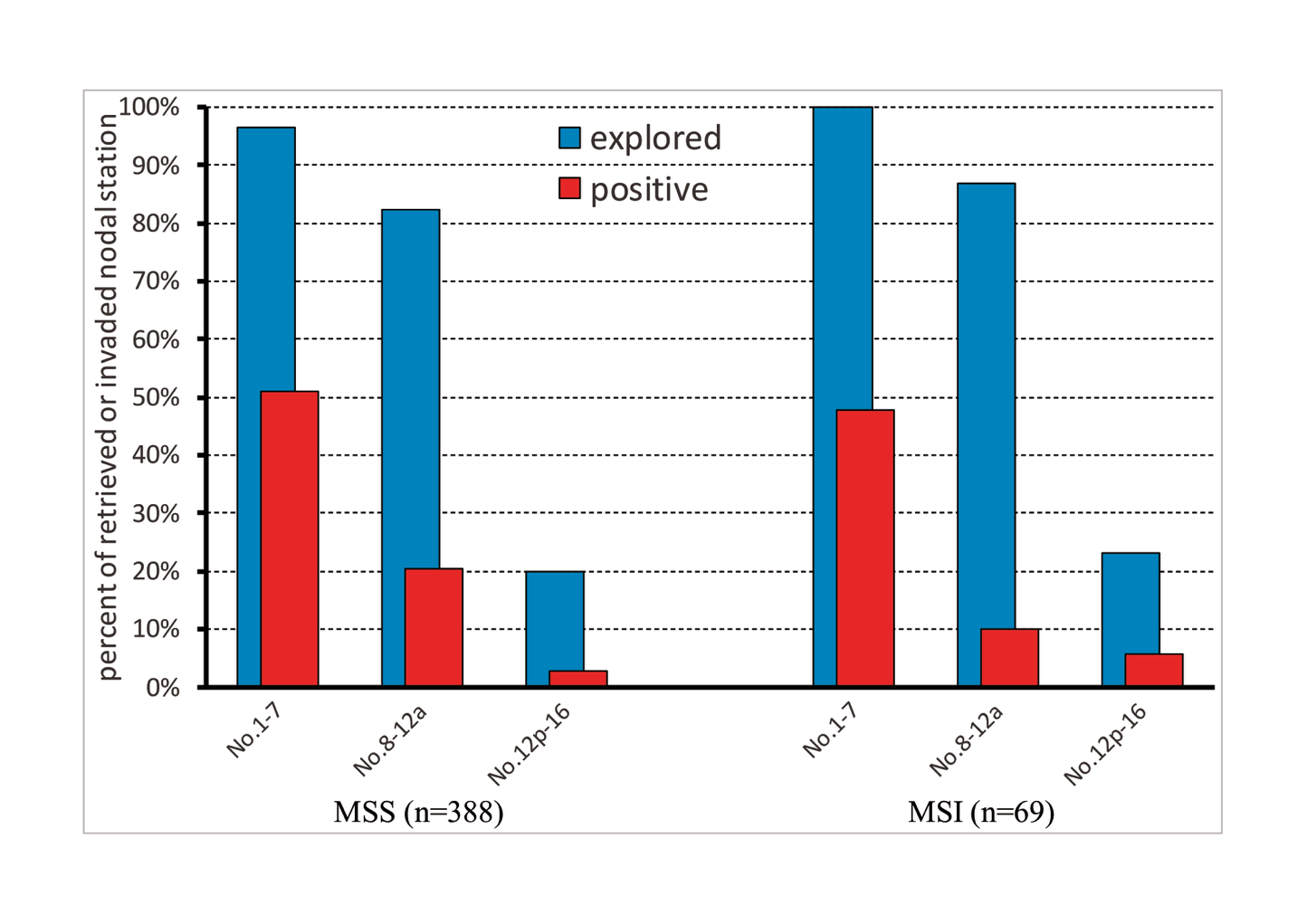
**

**Supplementary Table 1.** Distribution of Lauren’s histotypes across participating centers.

|  | Intestinal (n=479) | Diffuse (n=346) | Mixed (n=125) | P value |
| --- | --- | --- | --- | --- |
| Center |  |  |  | <0.001 |
| Stockholm | 71 (34%) | 94 (45%) | 42 (20%) |  |
| Amsterdam | 89 (67%) | 38 (29%) | 6 (5%) |  |
| Verona | 229 (49%) | 177 (38%) | 61 (13%) |  |
| Laikon | 50 (64%) | 19 (24%) | 9 (12%) |  |
| Ippokrateon | 40 (62%) | 18 (28%) | 7 (11%) |  |
